# Drug-associated psoriasis: a pharmacovigilance study based on the FAERS database

**DOI:** 10.3389/fphar.2026.1888496

**Published:** 2026-07-22

**Authors:** Sha Yi, Aimei Liu, Wuda Huoshen, Dengmei Xia, Xunmi Ma

**Affiliations:** 1 Department of Dermatology, Chengdu Integrated TCM & Western Medicine Hospital, Chengdu, Sichuan, China; 2 Department of Dermatology, The Second Affiliated Hospital of Chengdu Medical College, China National Nuclear Corporation 416 Hospital, Chengdu, Sichuan, China; 3 School of Stomatology, Southwest Medical University, Luzhou, Sichuan, China; 4 Department of Dermatology, West China Second University Hospital, Sichuan University, Chengdu, Sichuan, China

**Keywords:** disproportionality analysis, drug-associated psoriasis, FAERS database, pharmacovigilance, psoriasis

## Abstract

**Background:**

Drug-induced psoriasis has been increasingly recognized, but the spectrum of potential triggering medications remains incompletely characterized. We aimed to identify the drugs potentially associated with psoriasis using a large-scale and real-world pharmacovigilance analysis.

**Methods:**

Firstly, disproportionality analyses were used to detect psoriasis-related drug signals. Furthermore, Multivariable logistic regression and Bayesian shrinkage models were applied to evaluate the consistency of the detected associations after adjustment for available demographic and reporting-related factors. Finally, stratified analyses by sex, age, and reporter type were conducted to assess signal robustness.

**Results:**

A total of 30 drugs showed significant associations with psoriasis. These signals involved several pharmacological categories, including anti-infective agents (e.g., Abacavir, Fluconazole, Moxifloxacin, Sulfamethoxazole), anti-inflammatory and immunomodulatory drugs (e.g., Celecoxib, Tofacitinib), cardiovascular agents (e.g., Acebutolol, Telmisartan, Clonidine), neuropsychiatric drugs (e.g., Nortriptyline, Temazepam, Zopiclone, Wellbutrin), hormonal agents (e.g., Progesterone, Prometrium), and respiratory medications (e.g., Ventolin). Bayesian correction generally attenuated extreme effect estimates but confirmed the robustness of most signals. The stratified analysis also approve the above results.

**Conclusion:**

Our findings suggest that multiple drug classes are associated with psoriasis signals in pharmacovigilance data. These results highlight the importance of early recognition of drug-associated psoriasis and may assist clinicians in medication review and risk stratification in routine practice.

## Introduction

Psoriasis is a common chronic inflammatory skin disease, the etiology of psoriasis is multifaceted (Balak and Hajdarbegovic, 2017), and identifying factors that may trigger, induce, or aggravate the condition is significantly important for clinical practice (Tsankov et al., 2000).

Medication use represents a key concern, as an increasing number of drugs have been implicated in altering the course of psoriasis (Zheng et al., 2025; Ogawa and Izumi, 2025; Qureshi et al., 2024). Drug exposure may exacerbate existing psoriasis, induce psoriatic lesions on previously unaffected skin in patients with established disease, or even precipitate psoriasis in individuals without a personal or family history but who may be genetically susceptible (Tsankov et al., 2000). Rare studies have yet systematically examined this issue using large-scale real-world data.

The US Food and Drug Administration Adverse Event Reporting System (FAERS) represents an important resource for monitoring drug-related adverse reactions (Sakaeda et al., 2013). Signal detection analyses based on this database enable the preliminary identification of medications potentially associated with psoriasis.

In this study, we conducted a pharmacovigilance analysis using the FAERS database to identify potential drugs associated with psoriasis and provide real-world evidence on drug-related psoriasis. Logistic regression, time-to-onset (TTO) analysis, and sensitivity analyses were also incorporated to prioritize potential signals and characterize their temporal patterns, ultimately contributing to improved patient safety in clinical practice.

## Methods

### Study design

**FIGURE 1:**
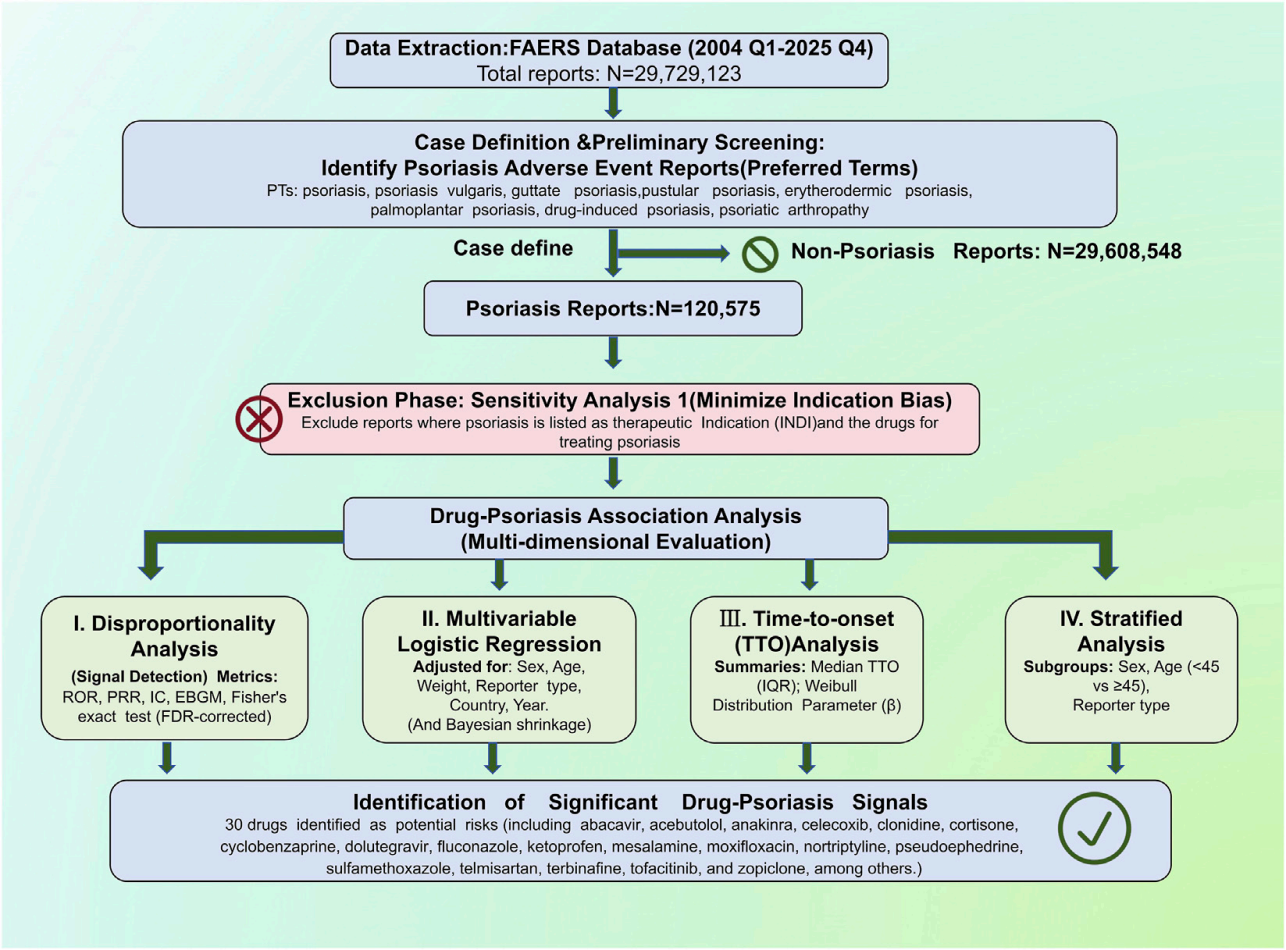
The flowchart of this study. FAERS, FDA Adverse Event Reporting System.

This study performed a retrospective pharmacovigilance analysis using the FAERS database to identify potential drug-psoriasis associations (Figure 1). FAERS data were retrieved from the publicly available Quarterly Data Extract Files released by the U.S. Food and Drug Administration. All reports submitted between the first quarter of 2004 and the fourth quarter of 2025 were included.

### Signal detection

Adverse event reports were obtained from the FAERS database from the first quarter of 2004 to the fourth quarter of 2025. Data from the DRUG and REAC tables were extracted for signal detection analyses. Psoriasis-related adverse events were identified using the Preferred Terms (PTs) “psoriasis,” “psoriasis vulgaris,” “guttate psoriasis,” and “pustular psoriasis,” “erythrodermic psoriasis,” “palmoplantar psoriasis,” and “drug-associated psoriasis”. Duplicate reports were identified using the CASEID field, and follow-up records were removed in accordance with FDA FAERS data processing recommendations. For each case, only the most recent report based on FDA_DT was retained. Drugs with role codes of primary suspect (PS) or secondary suspect (SS) were initially screened, and reports in which the drug was recorded as the primary suspect were included in the final analysis. Drug exposure information and psoriasis labels were merged at the individual case safety report level. Drug names were standardized by harmonizing brand and generic names, correcting spelling variations, and mapping to a curated drug dictionary to ensure consistency across reports. Combination products were decomposed into their constituent active ingredients, and each ingredient was analyzed individually in the primary analysis.

Disproportionality analyses were performed using a 2 × 2 contingency table, where “a” represented the number of reports involving both the target drug and psoriasis, “b” represented reports involving the target drug with other adverse events, “c” represented reports involving psoriasis with other drugs, and “d” represented reports involving other drugs with other adverse events (Bate and Evans, 2009).

Multiple signal detection metrics were calculated, including the reporting odds ratio (ROR), proportional reporting ratio (PRR), information component (IC), empirical Bayes geometric mean (EBGM), and Fisher’s exact test with false discovery rate (FDR) correction (Huoshen et al., 2026). Multiple testing correction was performed using the Benjamini–Hochberg false discovery rate (FDR) method, which is widely applied in pharmacovigilance signal detection studies. Statistical significance was defined as a *P*-value <0.05 after FDR adjustment. The criteria for signal detection were: a ≥100, the lower limit of the 95% confidence interval (CI) for ROR >1, PRR >2 with its lower CI limit >1, IC025 (the lower limit of IC) > 0, simplified estimate of EBGM05 (the lower limit of EBGM) > 2, and P-value <0.05 after FDR correction (Huoshen et al., 2026).

### Logistic regression analysis

To evaluate the independent associations between candidate drugs and psoriasis reports, multivariable logistic regression models were constructed using the FAERS database. Drug exposure was defined as a binary variable (1 = drug present in the report; 0 = absent). The outcome variable was the presence of psoriasis-related adverse events (Song et al., 2009). Models were adjusted for potential confounders, including sex group, age group, weight group, reporter type, reporting country, and report year. Reports with missing age information were retained and classified as a separate “Missing” category when applicable. The model was specified as follows:Logit [P (psoriasis report = 1)] = β_0_ + β_1_ × Drug Exposure + β_2_ × Sex + β_3_ × Age + β_4_ × Weight Group + β_5_ × Reporter Type + β_6_ × Country + β_7_ × Report Year Group (Huoshen et al., 2026).

Adjusted odds ratios (ORs) and 95% confidence intervals (CIs) were estimated using the “glm” function in R. Statistical significance was determined using two-sided tests with FDR correction.

To further improve estimate stability under sparse data conditions, Bayesian logistic regression was additionally performed using the bayesglm function from the “arm” package with weakly informative Cauchy priors.

### Time-to-onset analysis and weibull distribution analysis

Time-to-onset (TTO) was defined as the interval between the start date of suspected drug therapy (START_DT in the THER file) and the onset date of psoriasis-related adverse events (EVENT_DT in the DEMO file). Reports with incomplete or invalid date information were excluded, including records in which the event date preceded the drug initiation date. Time-to-onset analyses required complete and valid therapy start dates and event dates. Because date information was frequently missing in FAERS, the number of reports available for TTO analyses was substantially smaller than that used for signal detection. Formal TTO and Weibull analyses were restricted to drugs with at least 10 valid dated reports. The distribution of TTO was summarized using the median and interquartile range (IQR).

To characterize the temporal pattern of adverse event occurrence, the Weibull shape parameter (β) was estimated. The Weibull distribution is defined by a scale parameter (α) and a shape parameter (β), where β < 1 indicates early failure, β ≈ 1 random failure, and β > 1 wear-out failure.

### Sensitivity analysis

To improve the specificity of the analysis and reduce potential indication bias, several sensitivity analyses were performed. First, reports in which psoriasis was listed as the therapeutic indication (INDI) were excluded. This step was undertaken because psoriasis may be reported as an adverse event in patients who were already receiving treatment for psoriasis, which could lead to misclassification of the event as drug-associated. Second, reports involving medications commonly used for the treatment of psoriasis were excluded to further minimize confounding from underlying disease management. This restriction aimed to ensure that the identified cases more likely represented drug-associated psoriasis rather than the natural course or recurrence of the underlying disease.

These sensitivity analyses were conducted to assess the robustness of the signal detection results and to reduce potential biases related to indication and treatment effects.

As a supplementary analysis, drug names were additionally standardized to the active-ingredient level, and all signal-detection analyses were repeated after aggregating proprietary and generic names referring to the same active ingredient.

Drugs identified through signal detection were additionally categorized according to the Anatomical Therapeutic Chemical (ATC) classification system.

### Stratified analysis

To explore potential heterogeneity across patient subgroups, stratified analyses were conducted according to age, sex, and reporter type. Age was categorized into two groups (<45 years and ≥45 years). Sex was classified as male, female, or missing based on the FAERS database. Reporter type was defined according to the occupation code (OCCP_COD) and categorized as health professionals, consumers, and others or missing. Missing values were retained as separate categories rather than excluded from the analysis. Within each subgroup, disproportionality analyses were repeated to assess the consistency and robustness of the detected drug-psoriasis signals.

## Results

A total of 29,729,123 FAERS reports were included, comprising 120,575 psoriasis cases and 29,608,548 non-psoriasis reports (Table 1). Females accounted for a higher proportion among psoriasis reports compared with non-psoriasis reports (69.4% vs. 50.8%), while males represented 18.0% and 34.8%, respectively. Psoriasis cases were more frequently reported in individuals aged 18–64 years, particularly 45–64 years (28.4%), whereas patients aged ≥65 years were less represented (9.4% vs. 20.8% in non-psoriasis reports). Most psoriasis reports were submitted by health professionals (67.1%). Geographically, psoriasis reports were predominantly submitted from non-US countries (77.1%), while 54.0% of non-psoriasis reports originated from the United States. Reporting increased markedly in recent years, with the highest proportions observed during 2022–2024 (36.1%) and 2025 (24.7%).

**TABLE 1:** Clinical characteristics of patients reporting psoriasis events.

Characteristic	No psoriasis (N = 29608548)	Psoriasis (N = 120575)	*P value*
Gender, n (%)			
Male	10299579 (34.8%)	21658 (18.0%)	<0.001
Female	15029046 (50.8%)	83638 (69.4%)	​
Missing	4,279,923 (14.5%)	15,279 (12.7%)	​
Age, n (%)			
<18	1171792 (4.0%)	3412 (2.8%)	<0.001
18–44	3959721 (13.4%)	25653 (21.3%)	​
45–64	6085107 (20.6%)	34201 (28.4%)	​
≥65	6155645 (20.8%)	11323 (9.4%)	​
Missing	12236283 (41.3%)	45986 (38.1%)	​
Weight, n (%)			
<50	678953 (2.3%)	911 (0.8%)	<0.001
50–100	4439371 (15.0%)	23589 (19.6%)	​
>100	665214 (2.2%)	2222 (1.8%)	​
Missing	23825010 (80.5%)	93853 (77.8%)	​
Reporter, n (%)			
Health professional	15758485 (53.2%)	80873 (67.1%)	<0.001
Consumer	10963647 (37.0%)	34440 (28.6%)	​
Others	1417783 (4.8%)	194 (0.2%)	​
Missing	1468633 (5.0%)	5068 (4.2%)	​
Country, n (%)			
United States	15976663 (54.0%)	27541 (22.8%)	<0.001
Non-US	13565237 (45.8%)	92942 (77.1%)	​
Missing	66648 (0.2%)	92 (0.1%)	​
Report year, n (%)			
2004–2006	932637 (3.1%)	1268 (1.1%)	<0.001
2007–2009	1225216 (4.1%)	1657 (1.4%)	​
2010–2012	1780924 (6.0%)	2739 (2.3%)	​
2013–2015	3654049 (12.3%)	6896 (5.7%)	​
2016–2018	5513502 (18.6%)	11268 (9.3%)	​
2019–2021	7168166 (24.2%)	23493 (19.5%)	​
2022–2024	6798757 (23.0%)	43509 (36.1%)	​
2025	2534861 (8.6%)	29745 (24.7%)	​
Missing	436 (0.0%)	0 (0.0%)	​

Abbreviations: FAERS, FDA, adverse event reporting system.

### Signal detection

Disproportionality analyses were conducted to identify drugs potentially associated with psoriasis using the FAERS database. Reports in which psoriasis was recorded as the indication were excluded to minimize indication bias from psoriasis treatments. Signals were evaluated using four pharmacovigilance algorithms, including ROR, PRR, IC, EBGM and fisher test (Supplementary Table S1).

A total of 34 drugs met the predefined signal detection criteria across these methods, including abacavir, acebutolol, anakinra, celecoxib, clonidine, cortisone, cyclobenzaprine, dolutegravir, fluconazole, ketoprofen, mesalamine, moxifloxacin, nortriptyline, pseudoephedrine, sulfamethoxazole, telmisartan, terbinafine, tofacitinib, and zopiclone, among others (Supplementary Table S2). After ingredient-level standardization, 30 active ingredients met all predefined signal-detection criteria.

### Logistic regression analysis

**FIGURE 2:**
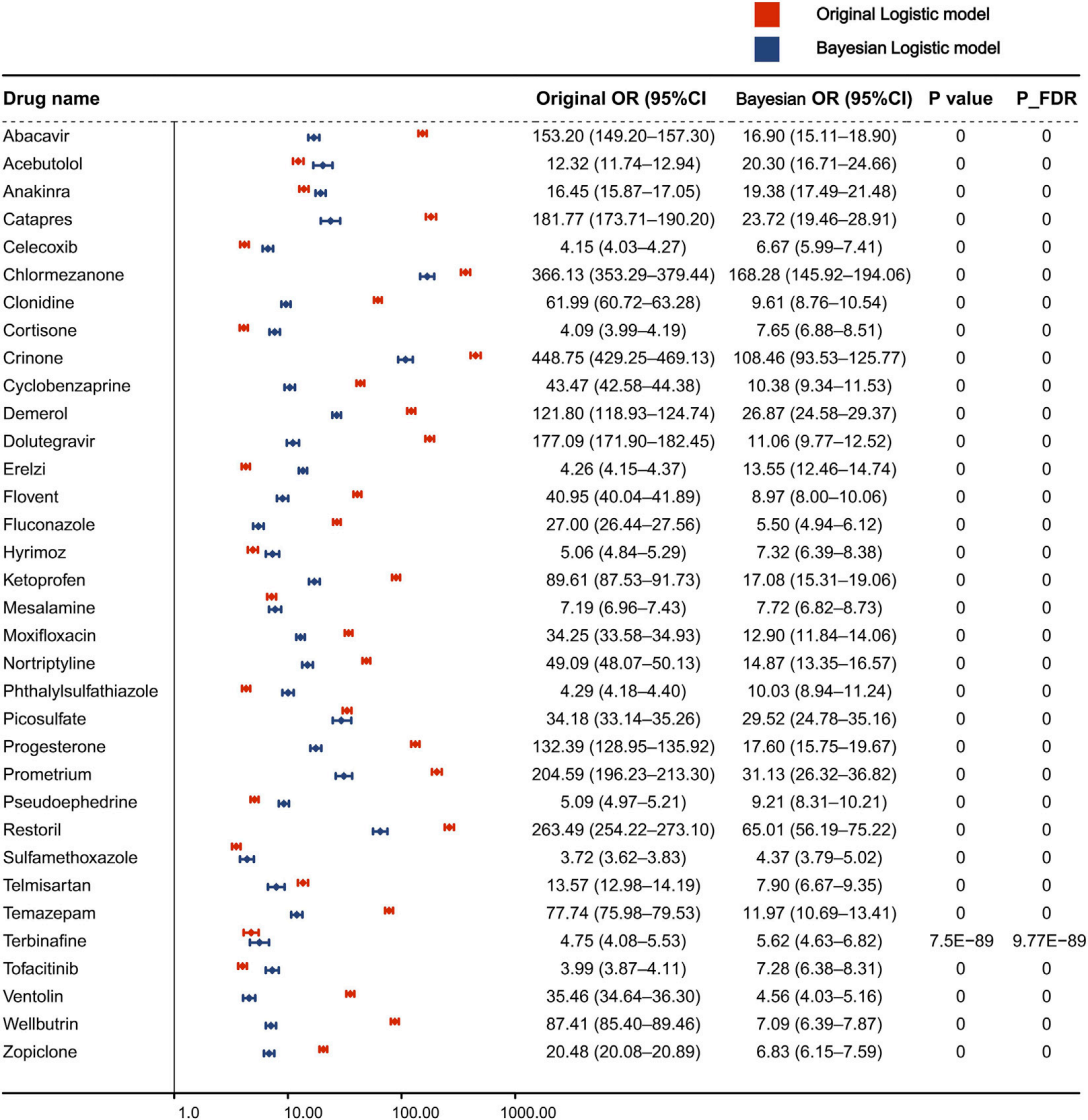
Forest plot of identified psoriasis‐related drugs after ROR, PRR, IC, EBGM, P value from Fisher’s exact test, sensitive analysis and logistic regression analysis. CI, confidence interval; OR, odds ratio.

Multivariable logistic regression analysis adjusting for sex group, age group, weight group, reporter type, reporting country, and report year identified significant associations between 34 drugs and psoriasis reports in the FAERS database (Figure 2). The adjusted odds ratios ranged from 3.72 to 448.75 in the primary logistic regression analysis. To reduce instability arising from sparse data and extreme coefficient estimates, Bayesian logistic regression was additionally performed using weakly informative priors. The shrinkage-adjusted estimates remained statistically significant after false discovery rate correction, with corrected ORs ranging from 4.37 to 168.28. Although the Bayesian model attenuated several extreme estimates, the overall signal pattern remained consistent with the primary analysis.

### Time-to-onset analysis and weibull distribution analysis

Time-to-onset (TTO) analysis was performed for drugs with sufficient reports. The median TTO varied across drugs, ranging from 17 days for terbinafine to 263 days for celecoxib (Table 2). Weibull distribution analysis indicated that most drugs exhibited an early failure pattern (β < 1), including erelzi (β = 0.77, 95% CI 0.61–0.97), hyrimoz (β = 0.80, 95% CI 0.65–0.97), and terbinafine (β = 0.61, 95% CI 0.40–0.92), suggesting that psoriasis events tended to occur shortly after drug initiation. In contrast, celecoxib (β = 0.93, 95% CI 0.59–1.48) showed a random failure pattern.

**TABLE 2:** Time-to-onset characteristics and weibull failure pattern of drug-induced psoriasis based on FAERS reports.

Drug	Cases	Median TTO (IQR), days	β (95% CI)	Failure pattern
Celecoxib	12	263 (258–776)	0.93 (0.59–1.48)	Random failure
Clonidine	1	1237 (1237–1237)	-	-
Erelzi	48	211 (53–520)	0.77 (0.61–0.97)	Early failure
Fluconazole	1	65 (65–65)	-	-
Hyrimoz	53	135 (35–212)	0.80 (0.65–0.97)	Early failure
Mesalamine	2	2 (2–2)	-	-
Telmisartan	1	279 (279–279)	-	-
Terbinafine	11	17 (3–22)	0.61 (0.40–0.92)	Early failure
Wellbutrin	3	8 (5–12)	-	-

Abbreviations: TTO, time to onset; IQR, interquartile range. β values were estimated using the Weibull distribution model. β < 1 indicates early failure type; β = 1 random failure; β > 1 wear-out failure. Drugs with <10 valid dated reports are shown for completeness only and were not considered informative for temporal pattern assessment.

### Stratified analysis

Stratified analyses by sex, age, and reporter type were conducted to evaluate the robustness of the detected signals. Overall, most drug-psoriasis associations remained consistent across subgroups. In age stratified analyses, tofacitinib, celecoxib, and sulfamethoxazole did not meet the EBGM signal criteria in the year group of ≥45, while mesalamine, terbinafine, telmisartan, and sulfamethoxazole were not positive in the year group of <45 (Supplementary Table S3).

In sex stratified analyses, several drugs, including ventolin, cortisone, fluconazole, wellbutrin, clonidine, cyclobenzaprine, sulfamethoxazole, and tofacitinib, did not show positive signals in males, whereas terbinafine did not reach signal thresholds in females (Supplementary Table S4). Progesterone-related signals remained positive in female-only analyses, indicating that these associations were not entirely explained by the female predominance of psoriasis reports in the overall dataset (Supplementary Table S4).

For reporter-type stratification, sulfamethoxazole was not positive among reports from health professionals, while wellbutrin, cortisone, ventolin, flovent, pseudoephedrine, sulfamethoxazole, and phthalylsulfathiazole were not positive in consumer-reported cases (Supplementary Table S5).

### Sensitivity analysis and ingredient-level standardization analysis

To further reduce potential indication bias, a sensitivity analysis was performed by excluding medications commonly used for psoriasis treatment, including biologics, targeted therapies, conventional systemic therapies, topical antipsoriatic agents, and related therapeutic products.

To evaluate whether signal inflation could arise from the use of proprietary drug names and formulation-specific entries in FAERS, an additional ingredient-level standardization analysis was performed. Drug names corresponding to the same active ingredient were aggregated and reanalyzed.

After ingredient-level standardization, 30 active ingredients met all predefined signal-detection criteria (Supplementary Table S6). The overall signal pattern remained largely consistent with the primary analysis, indicating that the detected associations were not driven by duplicate brand-name entries.

According to the ATC classification system, the detected signals were distributed across multiple therapeutic categories, including anti-infectives for systemic use (e.g., abacavir, fluconazole, moxifloxacin, and sulfamethoxazole), cardiovascular agents (e.g., acebutolol, clonidine, and telmisartan), nervous system drugs (e.g., nortriptyline, temazepam, and zopiclone), respiratory system drugs (e.g., pseudoephedrine and salbutamol), musculoskeletal system drugs (e.g., celecoxib and ketoprofen), hormonal preparations (e.g., progesterone and cortisone), alimentary tract and metabolism agents (e.g., mesalamine and picosulfate), and immunomodulating agents (e.g., anakinra, etanercept biosimilar, adalimumab biosimilar, and tofacitinib) (Supplementary Table S7).

## Discussion

In this large pharmacovigilance study based on the FAERS database, we systematically evaluated drugs potentially associated with psoriasis. Among more than 29 million reports, 120,575 psoriasis cases were identified, and 34 drugs met the predefined signal detection criteria across multiple pharmacovigilance algorithms. In addition, Multivariable logistic regression and Bayesian shrinkage analyses yielded findings that were broadly consistent with the disproportionality analyses. Finally, time-to-onset analysis suggested that psoriasis events associated with several drugs tended to occur early after treatment initiation.

Several biologically plausible mechanisms may explain the observed drug-psoriasis associations. According to the ATC classification system, the identified signals were distributed across multiple therapeutic categories, including anti-infectives for systemic use, cardiovascular agents, nervous system drugs, respiratory system drugs, hormonal preparations, musculoskeletal system drugs, alimentary tract and metabolism agents, and immunomodulating therapies.

Among conventional trigger drugs, anti-infective agents such as abacavir, dolutegravir, fluconazole, moxifloxacin, sulfamethoxazole, and terbinafine may contribute to psoriasis through immune dysregulation, hypersensitivity reactions, or altered T-cell activation (Tripathi et al., 2015; Mamkin et al., 2007). Cardiovascular agents including acebutolol, clonidine, and telmisartan have also been implicated, with β-blockers representing one of the most widely recognized drug classes associated with psoriasis exacerbation, potentially through reduced cyclic adenosine monophosphate signaling and altered keratinocyte proliferation (Tsankov et al., 2000; Kim and Del Rosso, 2010). Immunomodulatory therapies may also contribute to psoriasis development or exacerbation (Sieminska et al., 2024). Importantly, several identified agents, including anakinra, tofacitinib, and TNF inhibitors such as Erelzi and Hyrimoz, are not conventional psoriasis-triggering drugs. Instead, they are immunomodulatory therapies used to treat immune-mediated inflammatory diseases, including psoriasis. Therefore, the observed signals for psoriasis in association with these agents are more likely to reflect paradoxical psoriasis rather than classical drug-induced psoriasis. Paradoxical psoriasis is a recognized adverse event associated with biologic and targeted therapies, and paradoxical psoriasis is also thought to involve dysregulated interferon-α production by plasmacytoid dendritic cells, followed by activation of the IL-23/Th17 pathway (Collamer and Battafarano, 2010; Greuter et al., 2017). Although non-steroidal anti-inflammatory drugs (NSAIDs) such as celecoxib and ketoprofen are commonly used for their anti-inflammatory properties, they may paradoxically disrupt immune homeostasis in genetically or immunologically susceptible individuals. This imbalance may contribute to dysregulation of the IL-23/Th17 axis, which is central to psoriasis pathogenesis. Although the underlying mechanisms remain incompletely understood, these medications may influence neuroimmune pathways involved in psoriasis pathogenesis through modulation of neurotransmitter signaling and stress-response pathways (Gao et al., 2025; Yuda et al., 1991). Hormonal agents such as progesterone and cortisone may further affect immune homeostasis and inflammatory responses, although their precise roles in psoriasis require additional investigation. Nevertheless, the biological mechanisms underlying many of these signals remain incompletely understood. Given the inherent limitations of spontaneous reporting systems, the detected associations should be interpreted as pharmacovigilance signals rather than evidence of causality and require confirmation in future epidemiological and mechanistic studies.

The time-to-onset analysis provided additional insights into the temporal characteristics of drug-associated psoriasis. Most drugs demonstrated an early-failure pattern (β < 1), indicating that psoriasis events were more likely to occur shortly after drug initiation. This finding suggests that clinicians should remain vigilant during the early treatment period when prescribing medications potentially associated with psoriasis. Early recognition of drug-related psoriasis may facilitate timely drug withdrawal or therapeutic adjustment.

Stratified analyses by sex, age, and reporter type showed that most drug-psoriasis associations were generally consistent across subgroups, although some variability was observed. These differences may reflect variations in drug exposure patterns, immune responses, or reporting behaviors across patient populations. Nevertheless, the overall consistency of signals across subgroups supports the robustness of the detected associations.

This study has several limitations inherent to pharmacovigilance analyses using the FAERS database. First, spontaneous reporting systems are subject to underreporting and incomplete or missing clinical information, which may affect the accuracy of exposure and outcome assessment. Second, causal relationships cannot be established due to the lack of detailed patient-level data, including comorbidities, disease severity, and concomitant medications, and because residual confounding by indication and co-medication cannot be fully excluded, despite efforts to reduce bias by excluding reports in which psoriasis was listed as the treatment indication. Third, disproportionality analyses are vulnerable to reporting-related biases, including reporting bias, indication bias, and notoriety bias, and may yield unstable estimates for rarely reported drug-event pairs; although Bayesian shrinkage was applied to reduce extreme values, these associations should still be interpreted cautiously as hypothesis-generating signals rather than evidence of causality. Fourth, sex-related confounding may influence certain findings, particularly for progesterone-containing medications that are prescribed predominantly in women; however, signals persisted in female-only analyses, suggesting that the observed associations are unlikely to be fully explained by sex imbalance alone. Finally, the absence of signals for several drugs previously associated with psoriasis in clinical settings may reflect differences in reporting behavior, drug utilization patterns, and statistical thresholds inherent to spontaneous reporting systems, and should not be interpreted as evidence against established associations.

Despite these limitations, our study provides a comprehensive pharmacovigilance evaluation of drugs potentially associated with psoriasis using a large real-world dataset. The integration of multiple signal detection algorithms, regression analyses, and time-to-onset evaluation strengthens the reliability of the findings. Future epidemiological and mechanistic studies are warranted to confirm these associations and clarify the biological pathways underlying drug-associated psoriasis.

## Conclusion

In summary, this pharmacovigilance study identified multiple drugs across diverse therapeutic classes, including antiviral, anti-infective, anti-inflammatory, immunomodulatory, cardiovascular, and neuropsychiatric agents, that were associated with psoriasis reporting in the FAERS database. The detected signals were consistent across multiple disproportionality and regression analyses, and time-to-onset analyses indicated that many events occurred shortly after drug initiation. These findings may assist clinicians in early recognition of potential drug-associated psoriasis and support timely evaluation of suspected cases, including consideration of drug discontinuation or switching in clinically appropriate contexts. Further mechanistic and prospective studies are warranted to clarify the immunological and molecular pathways underlying these associations.

## Data Availability

The original contributions presented in the study are included in the article/Supplementary Material, further inquiries can be directed to the corresponding authors.
